# HIF-1α inhibits the TLR4/NF-κB signaling pathway and modulates intestinal flora in diarrhea-predominant irritable bowel syndrome

**DOI:** 10.1371/journal.pone.0355467

**Published:** 2026-08-06

**Authors:** Xing He, Dechun Wang, Bin Yang, Hezhong Yan, Honglin Ma, Haiqing Li, Jiaoxue Wang, Qian Gao, Senyuan Yu, Xilu Hou, Shicun Guo, Guobin Liao, Xiaowei Duan, Jun Tang

**Affiliations:** 1 Department of Gastroenterology, 901st Hospital of the PLA Joint Logistics Support Force, Hefei, Anhui Province, China; 2 Department of Gastroenterology, General Hospital of Xizang Military Command, Lhasa, Xizang, China; 3 The Third Stationed Outpatient Department, 901st Hospital of the PLA Joint Logistics Support Force, Hefei, Anhui Province, China; Emory University School of Medicine, UNITED STATES OF AMERICA

## Abstract

**Background:**

This study explored the regulatory effects of hypoxia-inducible factor-1α (HIF-1α) on the TLR4/NF-κB pathway and intestinal flora in diarrhea-predominant irritable bowel syndrome (IBS-D).

**Methods:**

Twenty-eight Wistar rats were randomized into four groups: model (n = 13), blank control (n = 5), HIF-1α upregulation (n = 5), and HIF-1α downregulation (n = 5). The IBS-D model group received combined acute and chronic stress stimulation for disease induction. On the basis of identical stress intervention, the HIF‑1α upregulation group was additionally exposed to a hypobaric hypoxic chamber, while the HIF‑1α downregulation group underwent intraperitoneal administration of the HIF‑1α antagonist 2‑methoxyestradiol to achieve targeted modulation of HIF‑1α expression.the control group was fed conventionally. Two model rats were euthanized on days 0, 7, 14, 21 to analyze colonic HIF-1α, TLR4 and NF-κB, and feces from the remaining 5 model rats were gathered on days 0, 7, 14, 21, 28 for 16S rRNA sequencing. Gastrointestinal symptoms, mucosal integrity and molecular expression were assessed after 28 days.

**Results:**

An IBS-D model was successfully established. HIF-1α expression in the model group increased progressively, while TLR4/NF-κB levels fluctuated but showed an overall increase. The levels of all three markers were significantly higher than those in the control group(P < 0.05). The HIF-1α downregulation group exhibited more severe IBS-D symptoms and higher TLR4/NF-κB expressions than the upregulation group (P < 0.05). At the genus level, the abundance of *Lachnospiraceae* NK4A136 decreased, whereas the abundance of the *Prevotellaceae* NK3B31 group, *Clostridia* UCG − 014, UCG − 005, and *Prevotellaceae* UCG − 001 increased (P < 0.05).

**Conclusion:**

Under systemic hypobaric hypoxic stress, HIF-1α suppresses the TLR4/NF-κB signaling pathway and alleviates intestinal inflammatory responses. Concurrent intestinal hypoxia and inflammatory injury jointly interfere with normal commensal colonization, accompanied by altered gut microbial composition in IBS-D model rats. Changes in HIF-1α levels are biologically correlated with shifts in intestinal microbiota, though direct causal regulation of gut flora by HIF-1α remains to be verified in further experiments.

## Introduction

Irritable bowel syndrome (IBS) is a chronic, recurrent functional gastrointestinal disorder with an unclear etiology and pathogenesis. It is primarily characterized by abdominal pain, diarrhea, and constipation. The global prevalence of IBS is estimated to be 11.2% [1]. This disorder is associated with recurrent symptoms, absence of specific treatments, significant patient distress, and substantial consumption of medical resources [2]. According to the Rome IV diagnostic criteria, IBS is classified into four subtypes based on bowel habits: diarrhea-predominant IBS (IBS-D), constipation-predominant IBS (IBS-C), mixed IBS (IBS-M), and unclassified IBS (IBS-U). Among these subtypes, IBS-D has the highest incidence and clinical impact [3]. Multiple factors, such as stress, intestinal infection, dysbiosis, and low-grade intestinal inflammation, contribute to its pathogenesis; however, the precise molecular mechanisms remain unclear [4].

Previous studies from this group [5] have demonstrated that stress induces low-grade inflammation through the TLR4/NF-κB signaling pathway, contributing to the development of IBS-D. However, the regulatory mechanisms underlying this pathway remain unclear. Hypoxia-inducible factor-1α (HIF-1α) is a transcription factor that is highly expressed in hypoxic environments and plays a critical role in maintaining intracellular homeostasis and facilitating tissue adaptation to hypoxic microenvironments [6]. In animal models of colitis, HIF-1α has been shown to reduce intestinal epithelial apoptosis by inhibiting NF-κB, thereby suppressing colonic inflammation [7]. Given the previous findings of elevated expression of both NF-κB and HIF-1α in IBS-D, the specific interaction between these two molecules in the pathogenesis of IBS-D requires further investigation. This study aimed to: (1) examine the dynamic relationship between HIF-1α and the TLR4/NF-κB pathway during the establishment of the IBS-D model; (2) determine the regulatory effects of HIF-1α upregulation and downregulation on TLR4/NF-κB in IBS-D rats.

## Materials and Methods

### 1. Animal Experiments

#### 1.1. Experimental Animals.

Twenty-eight specific pathogen-free (SPF) female Wistar rats, each weighing (160 ± 10) g, were obtained from Sibeifu (Beijing) Biotechnology Co., Ltd. (License No.: SCXK (Beijing) 2024−0001). The study was approved by the Ethics Committee of the 901st Hospital of the PLA Joint Logistics Support Force (Ethics Approval Number: XZ202301ZR0016G).

#### 1.2. Grouping and Treatment.

The rats were randomly assigned to four experimental groups:

1) IBS-D model group (*n* = 13): subjected to acute and chronic stress to induce IBS-D. Two rats were euthanized on days 0, 7, 14, and 21 to assess HIF-1α, TLR4, and NF-κB expression in colonic tissues. Fecal samples from the remaining five rats were collected on days 0, 7, 14, 21, and 28 for the analysis of intestinal flora. After 28 days of modeling, these five remaining rats were evaluated for gastrointestinal symptoms (1-hour stool volume, wet stool rate, intestinal sensitivity, sucrose water intake, and intestinal mucosal integrity. Colon tissues were collected for subsequent molecular and histological analyses.
2) Blank control group (*n* = 5): maintained under normal conditions without any treatment. After 28 days, the same assessments were performed as those in the IBS-D model group.
3) HIF-1α upregulation group (*n* = 5): exposed to a hypobaric hypoxic environment simulating an altitude of 3000–5000 m above sea level for 8 hours daily over 28 consecutive days, in combination with acute and chronic stress modeling.
4) HIF-1α downregulation group (*n* = 5): intraperitoneal injections of the HIF-1α inhibitor 2-methoxyestradiol (2ME2) at a dose of 5 mg/kg daily for 28 days in combination with acute and chronic stress modeling.

Rats in both the HIF-1α upregulation and downregulation groups underwent the same post-modeling assessments as in the IBS-D model group after 28 days.

#### 1.3. IBS-D Model Establishment.

The acute-chronic stress method [5] was employed, consisting of seven stressors administered randomly once per week, with no consecutive repetitions of the same stimulus. The stressors included cold stress (4°C for 3 min), horizontal vibration (120 cycles per minute for 40 min), water deprivation for 24 h, tail clipping for 1 min, continuous nocturnal lighting for 12 h, heat stress (45°C for 5 min), and food deprivation for 24 h. Following these procedures, rats were restrained by binding the front shoulder, forelimbs, and chest with paper tape for 1 h to limit forelimb movement, while allowing unrestricted overall activity except for head and face scratching.

#### 1.4. Model Validation Criteria.

Model success was evaluated using the following indicators [8–12]: 1h defecation volume, measured by the water avoidance stress method; wet stool rate, recorded using the filter paper imprint method; intestinal sensitivity, assessed by the abdominal withdrawal reflex (AWR) pressure threshold (2 points); sucrose water intake, measured as 1% sucrose water consumption within 1 h; and absence of organic lesions: determined by gross and microscopic examination of colon tissues for congestion, edema, erosion, or ulcers.

#### 1.5. Euthanasia and Sample Collection of Experimental Rats.

1)Rats were deeply anesthetized with isoflurane (3%−5%) and euthanized by cervical dislocation followed by immediate laparotomy. 3)The abdominal cavity was opened, and a 4 cm segment of the proximal sigmoid colon was excised, rinsed with normal saline, and examined for pathological changes. A 1 cm segment was preserved in liquid nitrogen for western blot analysis, while another segment was fixed in 10% formaldehyde for histological examination.

#### 1.6. Specific Euthanasia Criteria for Experimental Rats.

1)≥20% persistent weight loss + inability to take food/water for ≥24 hours (or food intake <50% normal for 2 days).2)Severe symptoms: unresponsive lethargy, mobility loss, dyspnea (respiratory rate >100/min), cyanosis, or pain behaviors (writhing, hunching, skin damage from grooming).3)Severe organ dysfunction (ALT > 300 U/L, AST > 400 U/L, BUN > 20 mmol/L, Cr > 150 μmol/L) or imaging-confirmed pathology.4)Uncontrollable local lesions (swelling/ulceration/necrosis) with unrelieved infection.Daily monitoring (twice) for prompt identification.The experimenters checked the animals twice a day (morning and evening) to monitor their physical condition and clinical symptoms.Specifically, the elapsed time between the confirmation of meeting the endpoint criteria and the implementation of euthanasia did not exceed 6 hours.

### 2. Detection methods

#### 2.1. Western blot analysis of HIF-1α, TLR4, and NF-κB.

Western blotting was performed as previously described [13] to detect the expression levels of HIF-1α, TLR4, NF-κB p65, MYD88, and GAPDH (loading control). Details of the primary and secondary antibodies used are provided in Table 1.

**Table 1:** Antibodies Used for Western Blot.

Antibody Name	Article Number	Company
HIF-1α	bs-20399R	Bioss
TLR4	35463	SAB
NF-κB P65	10745-1-AP	Wuhan Sanying
MYD88	23230-1-AP	Wuhan Sanying
GAPDH	AB-P-R 001	Hangzhou Xianzhi Biology
Goat Anti-Rabbit IgG with horseradish peroxidase (H + L)	A0208	Beyotime Biotechnology

**Abbreviations:** HIF-1α: Hypoxia-inducible factor 1-alpha; TLR4: Toll-like receptor 4; NF-κB P65: Nuclear factor kappa B P65; MYD88: Myeloid differentiation primary response gene 88; GAPDH: Glyceraldehyde 3-phosphate dehydrogenase; Goat Anti-Rabbit IgG (H + L): Goat Anti-Rabbit Immunoglobulin G (Heavy and Light chains) conjugated with horseradish peroxidase.

The experimental procedure included:Total protein was extracted from tissue samples, followed by quantification of protein concentrations. Equal quantities of total protein (20 μg) from each sample were loaded per lane for sodium dodecyl sulfate-polyacrylamide gel electrophoresis (SDS-PAGE). Subsequent experimental procedures included membrane electrotransfer, primary and secondary antibody incubation, chemiluminescence signal acquisition, and densitometric quantification of target protein bands.

#### 2.2. Hematoxylin-Eosin (HE) staining of colon tissues.

Colon tissues fixed in formalin were dehydrated, embedded in paraffin, sectioned, and stained with HE [14]. The integrity of the sigmoid mucosal epithelium and the presence of defects, erosion, edema, or ulcers were evaluated using an Olympus light microscope at 40 × magnification.

#### 2.3. 16S rRNA sequencing of fecal flora.

Fecal microbial DNA was extracted using the TIANamp Stool DNA Kit (TIANGEN, China). DNA purity and concentration were assessed via 1% agarose gel electrophoresis and quantified using a NanoDrop™ 2000 spectrophotometer (Thermo Scientific, USA) [15]. The V3–V4 hypervariable region of the bacterial 16S rRNA gene was amplified by polymerase chain reaction (PCR) with the primer pairs 341F (5′‑CCTACGGGNGGCWGCAG‑3′) and 806R (5′‑GGACTACHVGGGTWTCTAAT‑3′). Each sample was amplified in triplicate to ensure reproducibility. Purified PCR products were obtained using the AxyPrep DNA Gel Recovery Kit (Axygen, USA), and the final DNA concentration was determined with a QuantiFluor™‑ST fluorometer (Promega, USA). Amplicon libraries were constructed and sequenced on the Illumina MiSeq platform (Illumina, USA). Raw sequencing reads were quality-filtered, denoised, and chimeric sequences were removed using the DADA2 pipeline embedded in QIIME2 to generate high‑quality amplicon sequence variants (ASVs). Taxonomic annotation of ASVs was performed against the SILVA v138 reference database with the QIIME2 naïve Bayes classifier, which generates taxonomic labels including UCG and NK placeholder groups for unclassified microbial taxa.

### 3. Statistical analysis

Statistical analyses were conducted using SPSS version 22.0. Normality was assessed before analysis. Measurement data are presented as mean ± standard deviation (SD), and intergroup comparisons were performed using independent samples t-tests. A *P*-value < 0.05 was considered statistically significant.

## Results

### 1. Successful establishment of the IBS-D rat model

**Fig 1:**
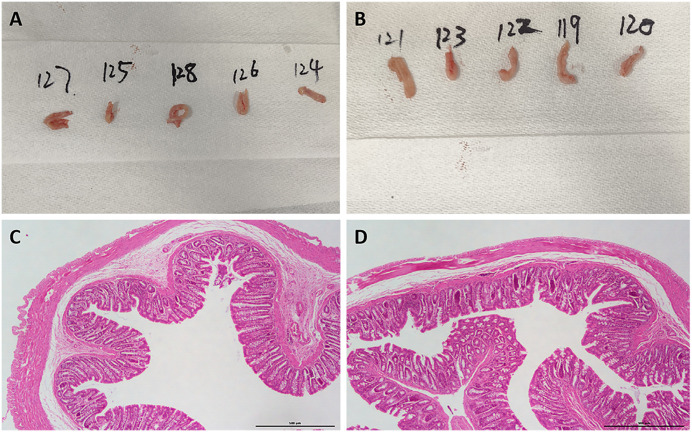
Gross and histopathological features (HE staining, × 40) of intestinal tissues in control and IBS-D model rats. Note: (1A, C) Gross morphology and HE staining patterns of intestinal tissues from the control group; (1B, D) Gross morphology and HE staining patterns of intestinal tissues from the IBS-D model group. No significant intestinal mucosal hyperemia, edema, erosion, or ulceration was observed in either group (HE staining, × 40).

No experimental rats died unexpectedly prior to meeting the preset humane endpoint criteria. In post-modeling, the 1h defecation volume and wet stool rate in the model group were significantly higher than those in the blank control group (*P* < 0.05). In contrast, the AWR pressure threshold and sucrose water intake were significantly lower (*P* < 0.05, Table 2). Gross and HE staining of colon tissues showed no evidence of congestion, edema, erosion, or ulcers in the model group (Fig 1).Raw data of relevant indicators throughout model establishment procedures are summarized in S1 Table.

**Table 2:** Comparison of Gastrointestinal Indicators Between the Model and Control Groups.

Group	n	Defecation Volume (grain/h or time/h)	Wet Stool Rate (%)	AWR (2 score)(mmHg)	Sucrose Water Intake (ml)
**Model group**	5	10.2 ± 2.17^a^	21.42 ± 9.48^a^	25 ± 3.54	9 ± 1.82
**Control group**	5	5.8 ± 1.48	4.16 ± 2.78	37 ± 4.72	14.2 ± 1.48
**t-value**		3.746	3.907	4.548	4.957
***P*-value**		0.006	0.005	0.002	0.001

**Note:** ^a^ *P* < 0.05 vs. control group.

### 2. Dynamic changes of HIF-1α, TLR4, and NF-κB during modeling

**Fig 2:**
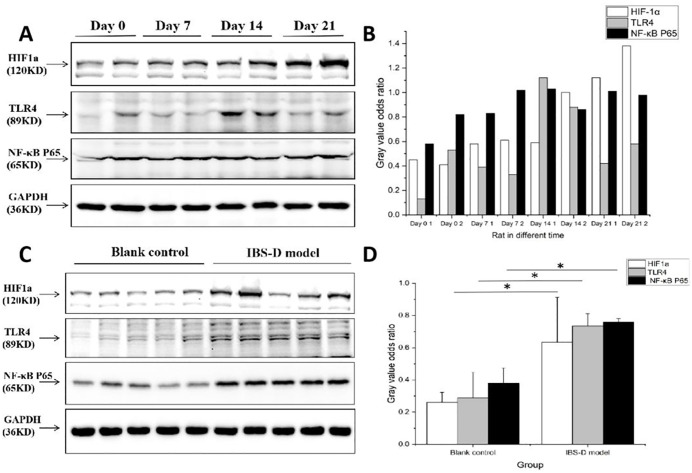
The temporal dynamics of HIF-1α, TLR4, and NF-κB expression during IBS-D model establishment. Note: (2A, B) Densitometric analysis and gray value ratios from western blot experiments demonstrated sustained upregulation of HIF-1α expression during model construction. In contrast, TLR4 and NF-κB expression fluctuated but showed an overall increasing trend. (2C, D) Western blot-based densitometry and gray value ratio assessments confirmed that the expression levels of HIF-1α, TLR4, and NF-κB were significantly higher in the IBS-D model group than in the blank control group (*P  <  0.05).

In the model group, HIF-1α expression increased steadily from day 0 to day 21, whereas TLR4 and NF-κB expression fluctuated but demonstrated an overall upward trend (Fig 2A, 2B). In post-modeling, the levels of HIF-1α, TLR4, and NF-κB in the model group were significantly higher than those in the blank control group (*P* < 0.05, Fig 2C, 2D).The full-length, uncropped western blot bands are presented in S1 Fig.

### 3. Effects of HIF-1α Modulation on IBS-D Symptoms

The HIF-1α downregulation group demonstrated significantly increased defecation volume and wet stool rate, as well as lower AWR pressure threshold and sucrose water intake, compared to the HIF-1α upregulation group (*P* < 0.05, Table 3). These findings suggest that the HIF-1α downregulation group experienced more severe IBS-D symptoms.Raw data of relevant indicators throughout model intervention procedures are summarized in S1 Table.

**Table 3:** Comparison of IBS-D Symptoms Between HIF-1α Upregulation and Downregulation Groups.

Group	n	Defecation Volume (grain/h or time/h)	Wet Stool Rate (%)	AWR (2 score)(mmHg)	Sucrose Water Intake (ml)
**Low HIF-1α group**	5	11.6 ± 4.05^a^	22.72 ± 10.23^a^	21 ± 2.07	7.6 ± 1.14
**High HIF-1α group**	5	6.8 ± 1.92	4.82 ± 2.98	26 ± 2.70	11.8 ± 1.48
**t-value**		2.395	3.756	3.286	5.027
***P*-value**		0.044	0.006	0.011	0.001

**Note:** ^a^ *P* < 0.05 vs. HIF-1α upregulation group.

### 4. Regulatory Effects of HIF-1α on TLR4 and NF-κB

**Fig 3:**
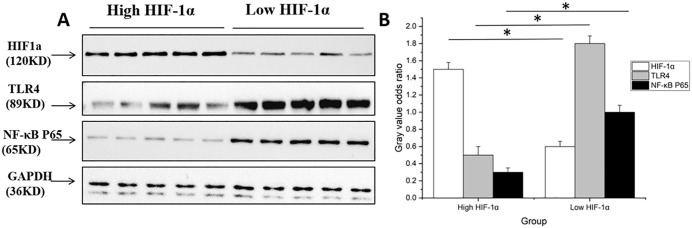
Western blot analysis of HIF-1α, TLR4, and NF-κB expression differences between the high and low HIF-1α groups. Note:(3A) Western blot densitometric analysis showed increased HIF‑1α but decreased TLR4 and NF‑κB protein abundance in the high‑HIF‑1α group versus the low‑HIF‑1α group.(3B) Relative gray value quantification showed significantly higher HIF‑1α expression, yet remarkably lower TLR4 and NF‑κB abundance, in the HIF‑1α upregulated group relative to the HIF‑1α downregulated group.(*P  <  0.05).

HIF-1α expression was significantly higher in the upregulation group than in the downregulation group (*P* < 0.05), indicating effective modulation of HIF-1α. In contrast, TLR4 and NF-κB expression levels were significantly reduced in the HIF-1α upregulation group (*P* < 0.05, Fig 3), suggesting that HIF-1α inhibits the TLR4/NF-κB signalling pathway in rats with IBS-D.The full-length, uncropped western blot bands are presented in S1 Fig.

### 5. Dynamic changes of intestinal flora during modeling

**Fig 4:**
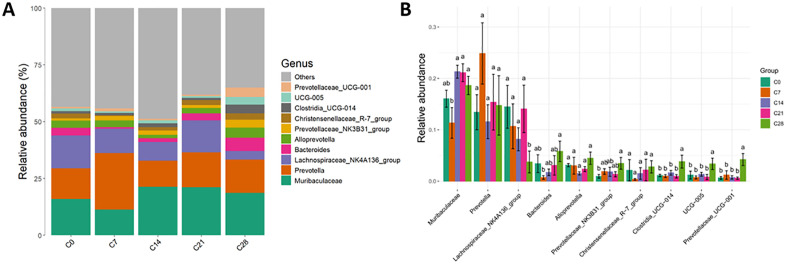
Temporal dynamics of intestinal microbiota at the genus level in rats at 0, 7, 14, 21, and 28 days post-modeling. Note:(4A) Genus-level species composition analysis indicated a decreasing trend in the relative abundance of Lachnospiraceae NK4A136 group, whereas other intestinal microbial taxa exhibited increasing trends. (4B) Comparative analysis of genus-level species expression differences showed a statistically significant increase in Prevotellaceae NK3B31 group, Clostridia UCG− − 014, UCG− − 005, and Prevotellaceae UCG− − 001, and a statistically significant decrease in the NK4A136 group (Pa/b  <  0.05).

Analysis of 16S rRNA sequencing data revealed significant changes in the intestinal flora composition at the genus level during the modeling process. The abundance of *Lachnospiraceae* NK4A136 group decreased progressively (*P* < 0.05), whereas the abundance of the *Prevotellaceae* NK3B31 group, *Clostridia* UCG − 014, UCG − 005, and *Prevotellaceae* UCG − 001 increased (*P* < 0.05, Fig 4).

## Discussion

Establishing a valid IBS-D animal model is essential for this study. As a functional gastrointestinal disorder, IBS-D requires a model that simulates multiple pathogenic factors. An acute-chronic stress protocol was employed, resulting in a model that aligned with the clinical characteristics of IBS-D, as confirmed by assessments of gastrointestinal symptoms and intestinal mucosal integrity.

Previous studies have identified elevated expressions of HIF-1α and the TLR4/NF-κB pathway in IBS-D; however, the dynamic changes during pathogenesis remain unclear. Serial sampling during the modeling process in this study demonstrated a continuous linear increase in HIF-1α expression, which is closely linked to stress-induced systemic hypoxia [16]. HIF-1α contributes to intracellular homeostasis by regulating hypoxia-responsive genes, enhancing tolerance to tissue hypoxia, and inhibiting intestinal epithelial apoptosis [17,7]. These findings suggest a protective role in IBS-D by sustaining low-grade intestinal inflammation [18].

In contrast, TLR4 and NF-κB expression levels fluctuated but increased overall during modeling, consistent with previous findings that the TLR4/NF-κB pathway mediates stress-induced low-grade inflammation in IBS-D [5,19–21]. The observed non-linear increase in TLR4/NF-κB, along with the lack of parallelism with HIF-1α, suggests that HIF-1α may inhibit rather than activate the TLR4/NF-κB pathway, thereby limiting the progression of intestinal inflammation.

To confirm this regulatory relationship, HIF-1α expression was modulated via hypobaric hypoxia or pharmacological inhibition [22]. Rats with downregulated HIF-1α exhibited more severe IBS-D symptoms and increased TLR4/NF-κB expressions, whereas HIF-1α upregulation alleviated symptoms and suppressed the TLR4/NF-κB pathway. These findings indicate that HIF-1α exerts a protective effect in IBS-D by inhibiting the TLR4/NF-κB-mediated inflammatory response.

Intestinal dysbiosis represents a hallmark of IBS‑D, yet its regulatory mechanisms have not been fully clarified [23,24]. Our stress-induced IBS‑D model without direct microbial intervention yielded prominent gut microbial perturbations. The *Lachnospiraceae* NK4A136 group, a biomarker for mood disorders [25], was depleted, consistent with diminished sucrose preference (anhedonia-like behavior) and the high comorbidity of IBS‑D with anxiety and depression [26].

*Prevotellaceae* UCG‑001, a fiber-fermenting taxon generating short-chain fatty acids (SCFAs), participates in metabolism, immune modulation and gut–brain axis homeostasis. Existing evidence confirms its anti-inflammatory capacity via suppressing pro-inflammatory cytokines (IL-6, IL-8, TNF-α) and facilitating colonic tissue repair [27,28]. In our study, its abundance gradually elevated alongside disease progression, which we postulate as a compensatory feedback response counterbalancing intestinal inflammation to sustain the low-grade inflammatory phenotype typical of IBS‑D. Several other genera (*Clostridia* UCG-014, UCG-005) also showed upward trends, collectively implicating reshaped microbiota in IBS‑D pathogenesis.

We propose that stress-triggered intestinal hypoxia (which facilitates anaerobe proliferation) and TLR4/NF-κB-mediated low-grade inflammation synergistically disturb commensal colonization and drive dysbiosis. Further validation is required to dissect the precise crosstalk between hypoxia, inflammation and gut microbiota.

## Conclusion

Under systemic hypobaric hypoxic stress, HIF‑1α levels are closely correlated with the activity of the TLR4/NF‑κB signaling pathway during IBS‑D progression. Relative to the HIF‑1α downregulation group, the HIF‑1α overexpression group displayed reduced TLR4 and NF‑κB expression and attenuated intestinal inflammatory responses, which helped maintain the mild low‑grade inflammatory state observed in IBS‑D rats. Concurrent intestinal inflammatory stimulation and tissue hypoxia disrupt normal intestinal commensal colonization, ultimately accompanying gut microbiota dysbiosis in IBS‑D model rats. Changes in HIF‑1α expression coincide with shifts in gut microbial composition under systemic hypobaric hypoxic conditions; however, direct causal effects of HIF‑1α on modulating intestinal flora cannot be confirmed based on the current dataset and remain to be characterized in follow-up functional experiments.

## Supporting information

S1 Fig The full-length, uncropped western blot bands. (PDF)

S1 Table Raw data of relevant indicators throughout model establishment and intervention procedures. (XLSX)
