# Supplementary material for: HIF-1α inhibits the TLR4/NF-κB signaling pathway and modulates intestinal flora in diarrhea-predominant irritable bowel syndrome
Source: PLoS One. 2026 Aug 6;21(8):e0355467. doi: 10.1371/journal.pone.0355467 (PMC13446686; doi:10.1371/journal.pone.0355467)

Figure 2 A HIF1α

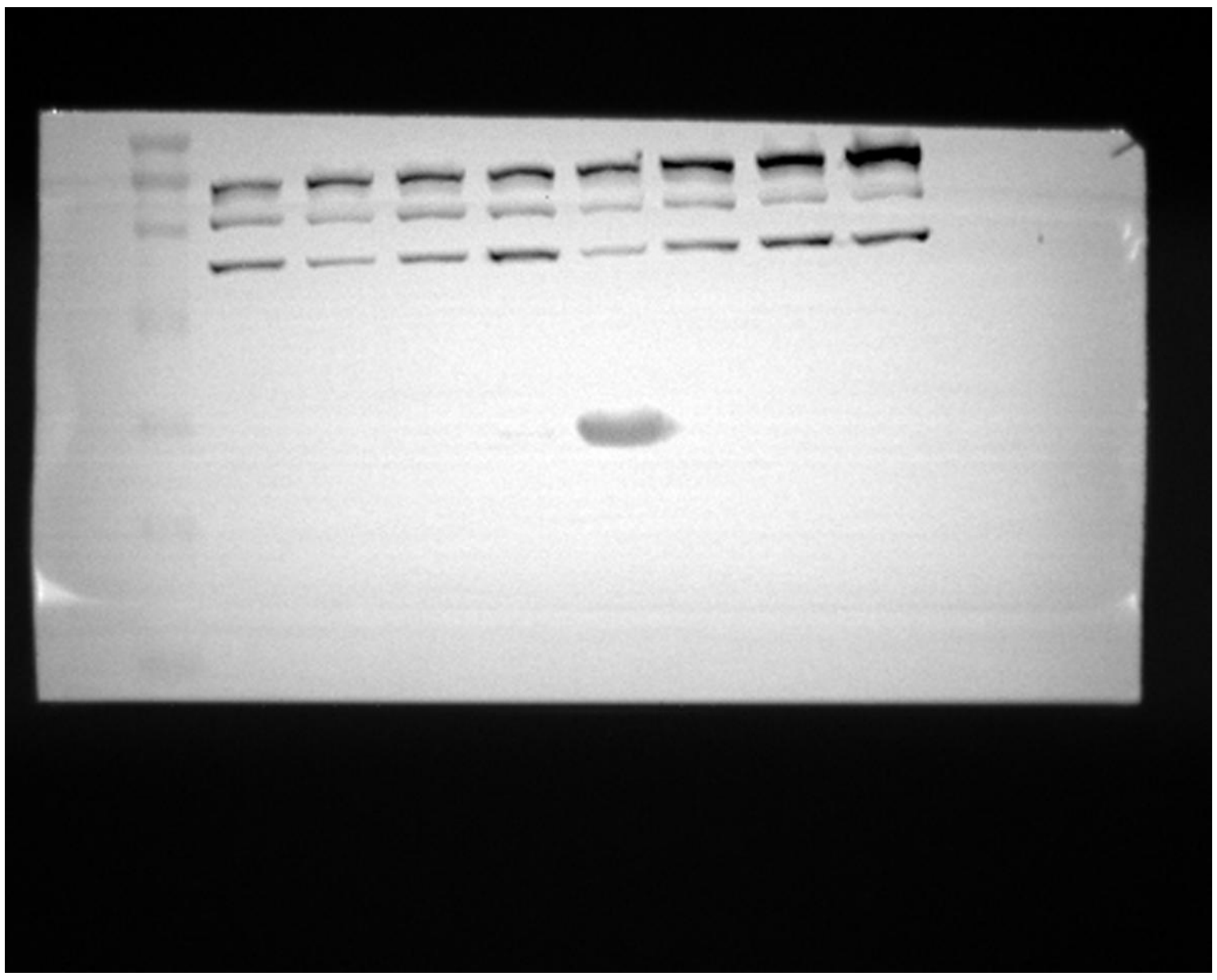

Figure2A TLR4

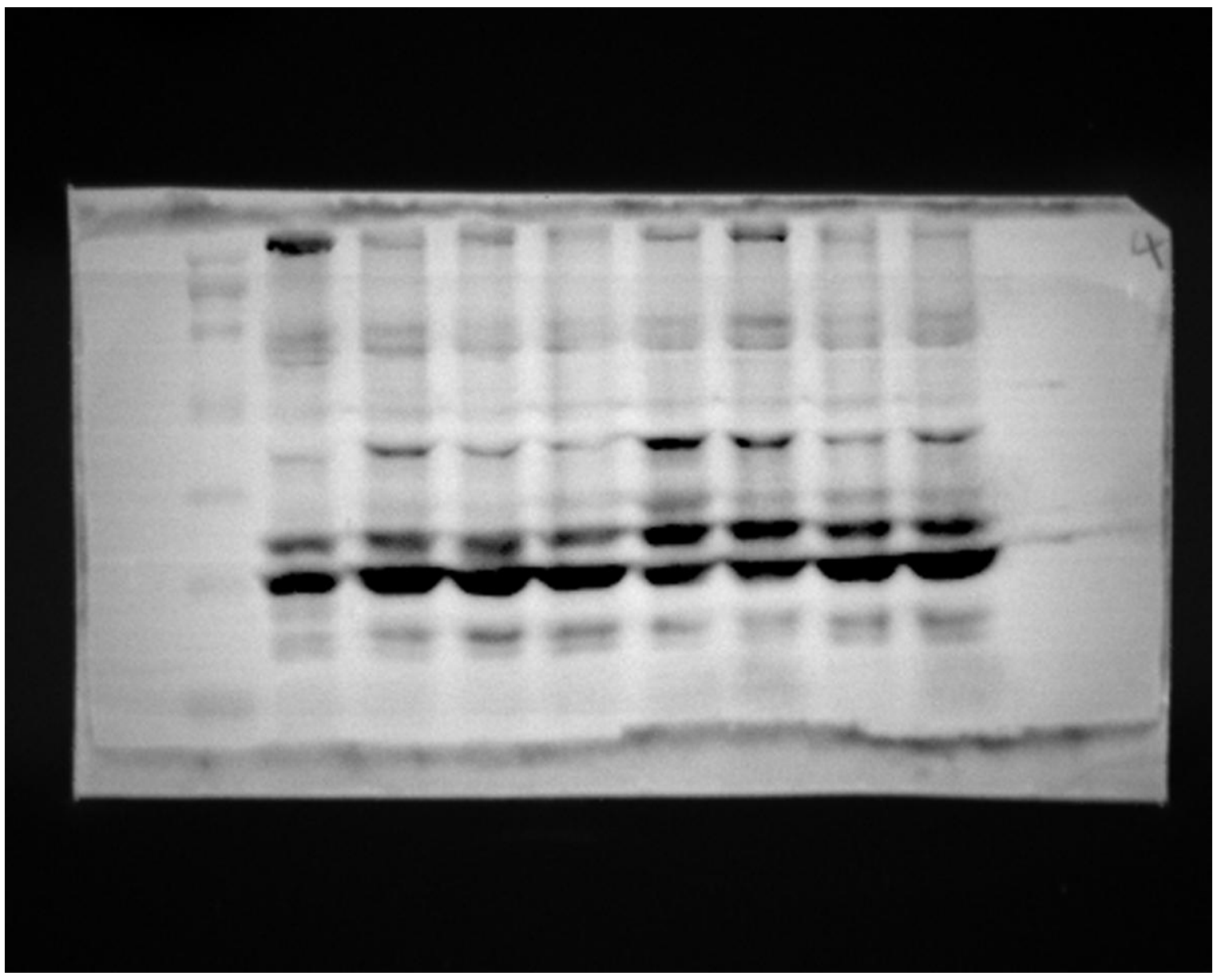

Figure2A GAPDH

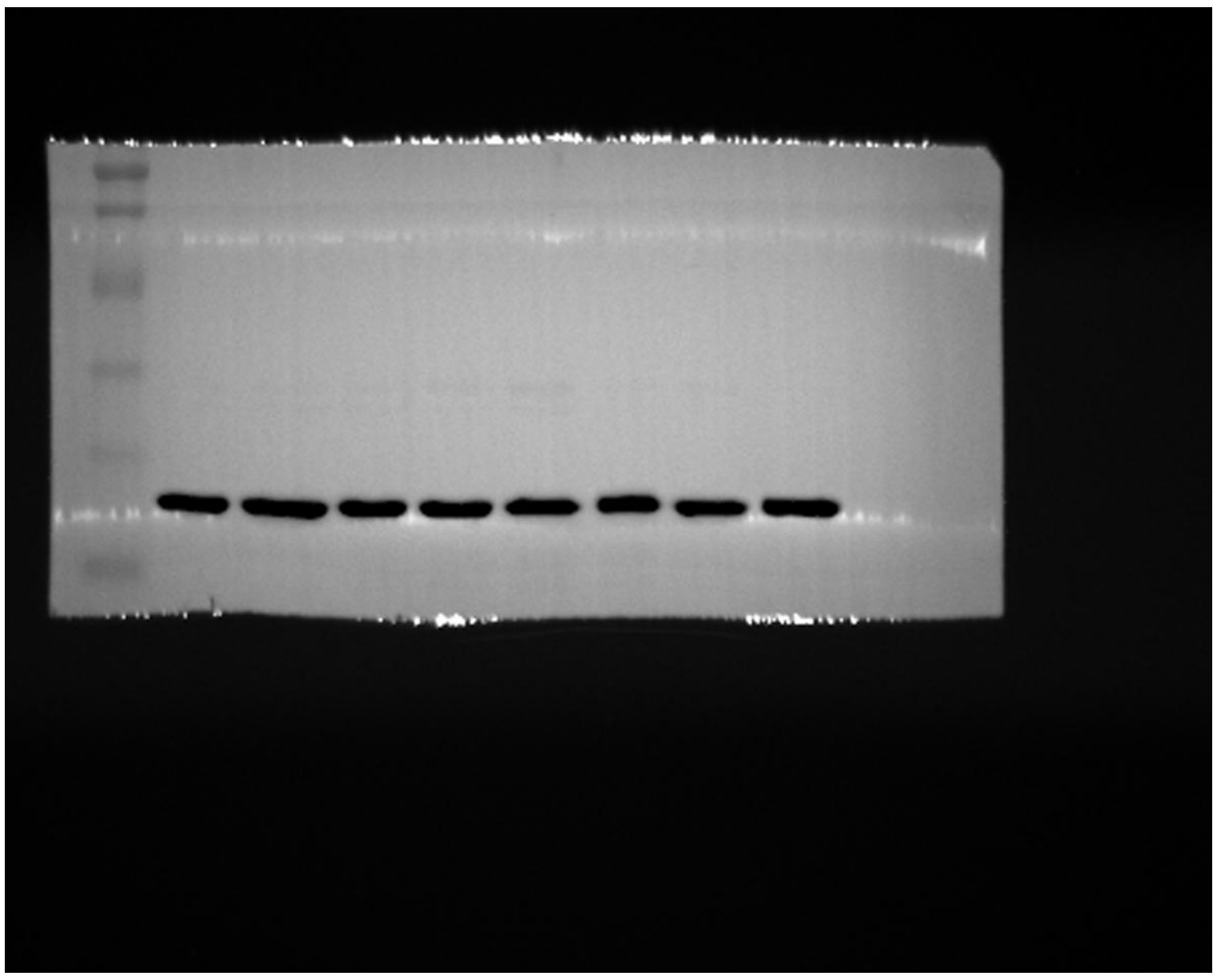

Figure 2 A NF-κB

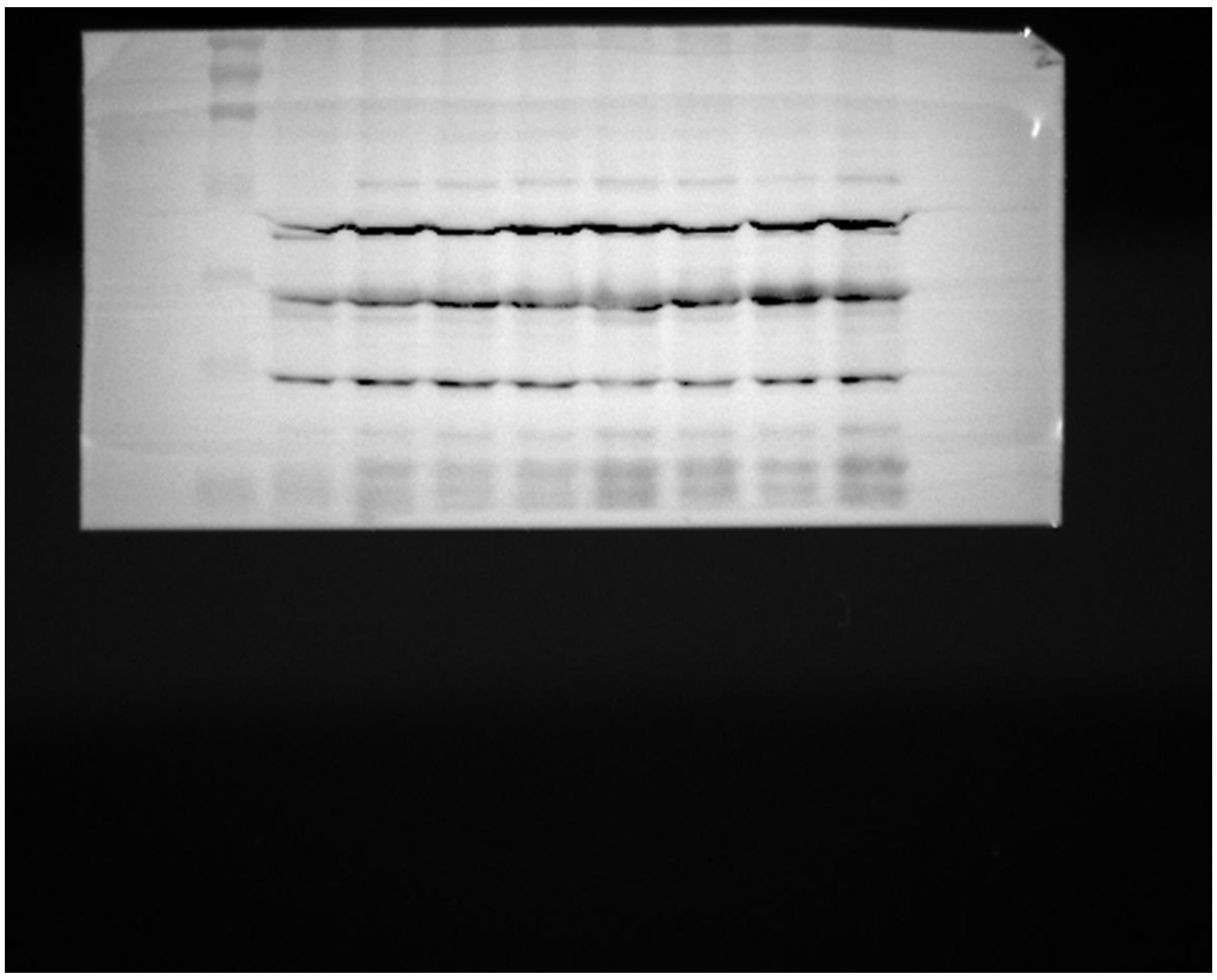

Figure2C HIF1α

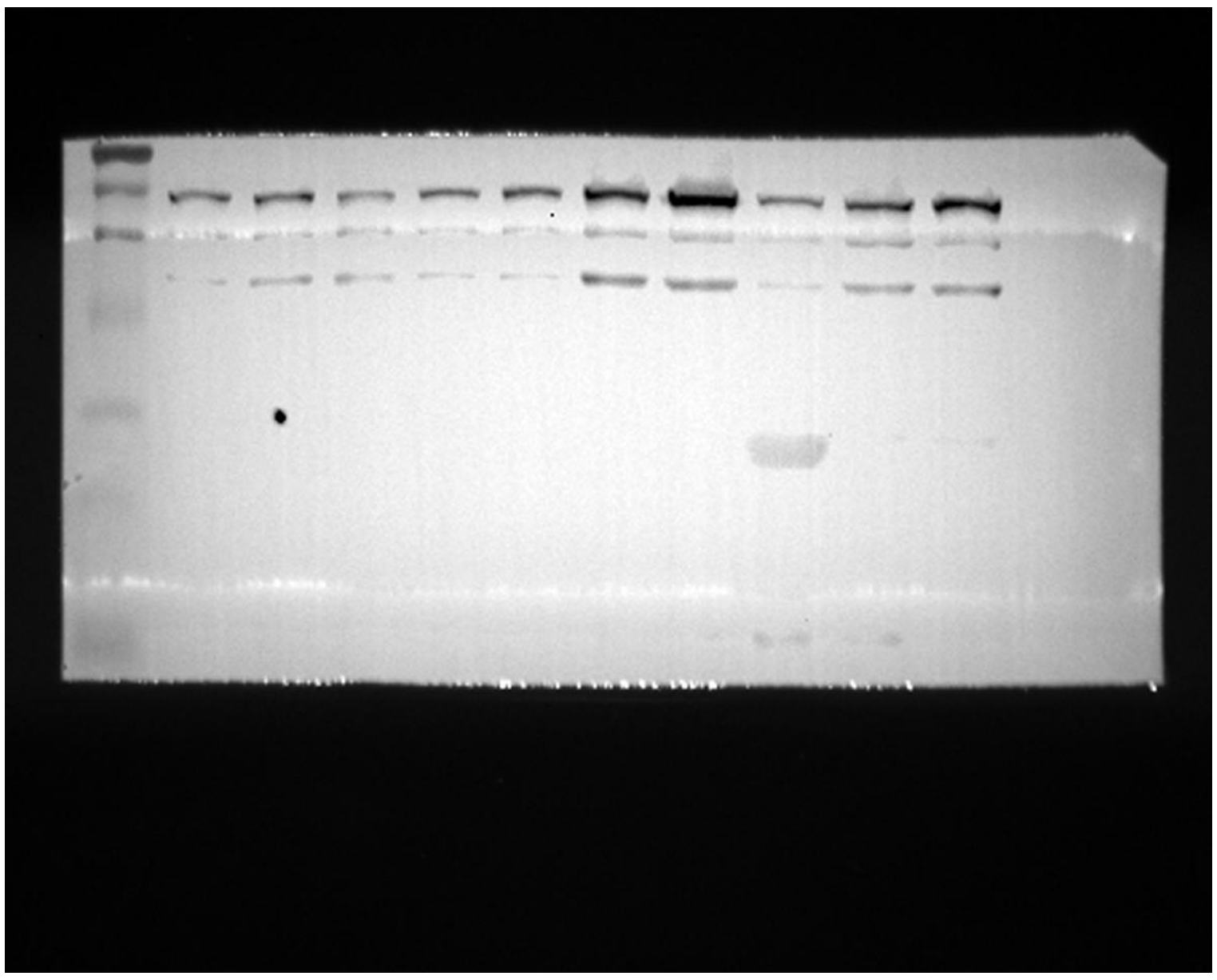

Figure 2 C TLR 4

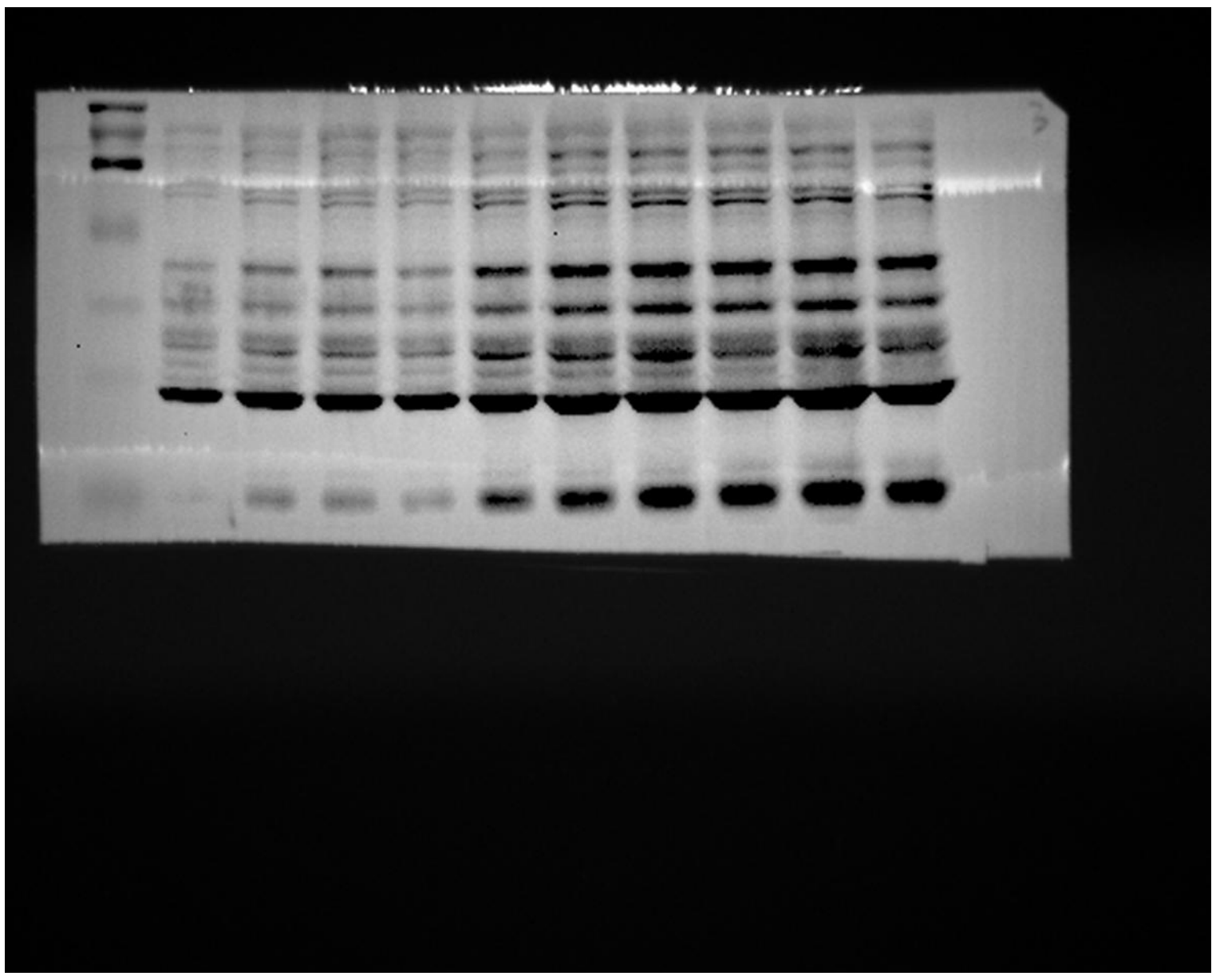

Figure 2 C G A P D H

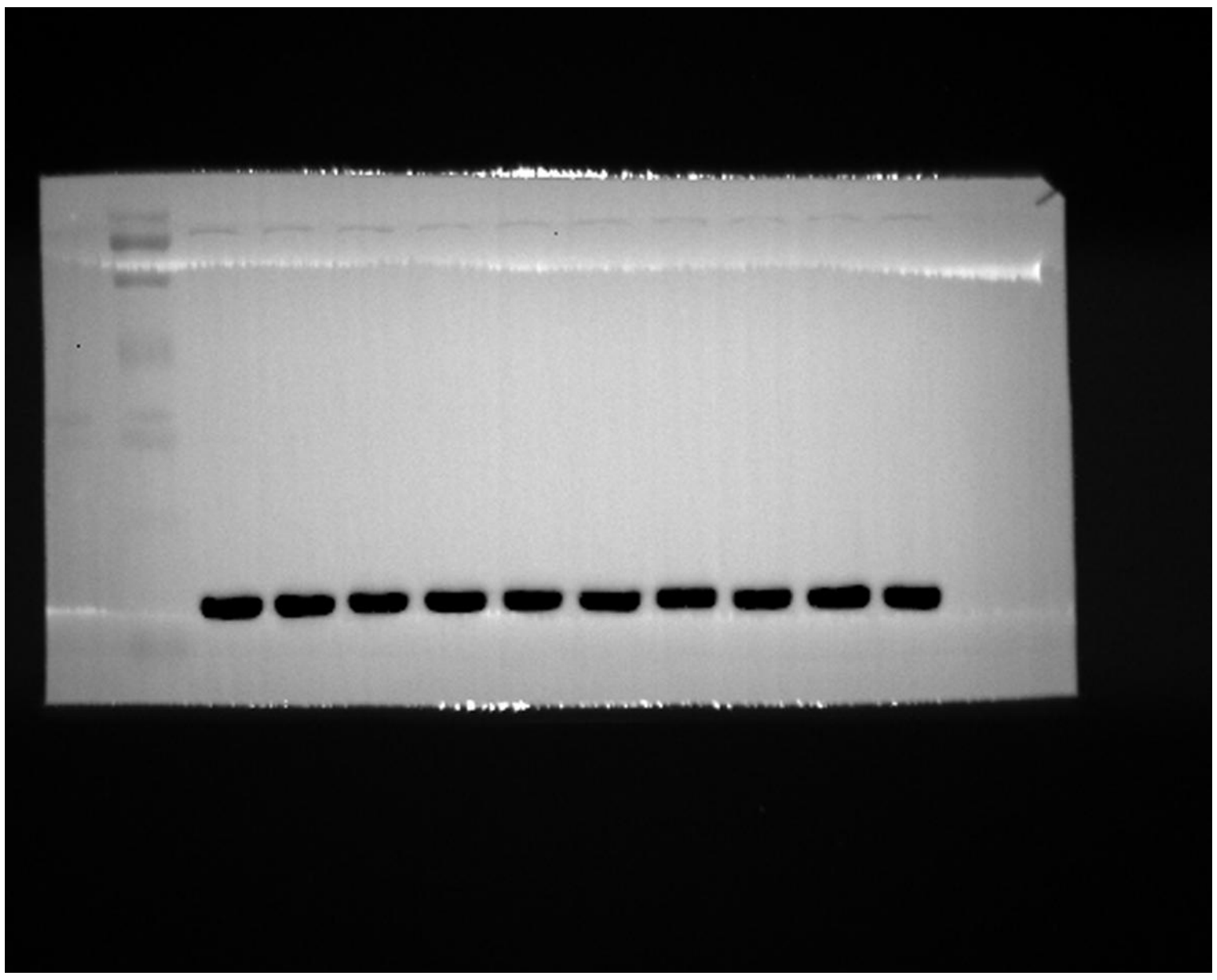

Figure2C NF-κB

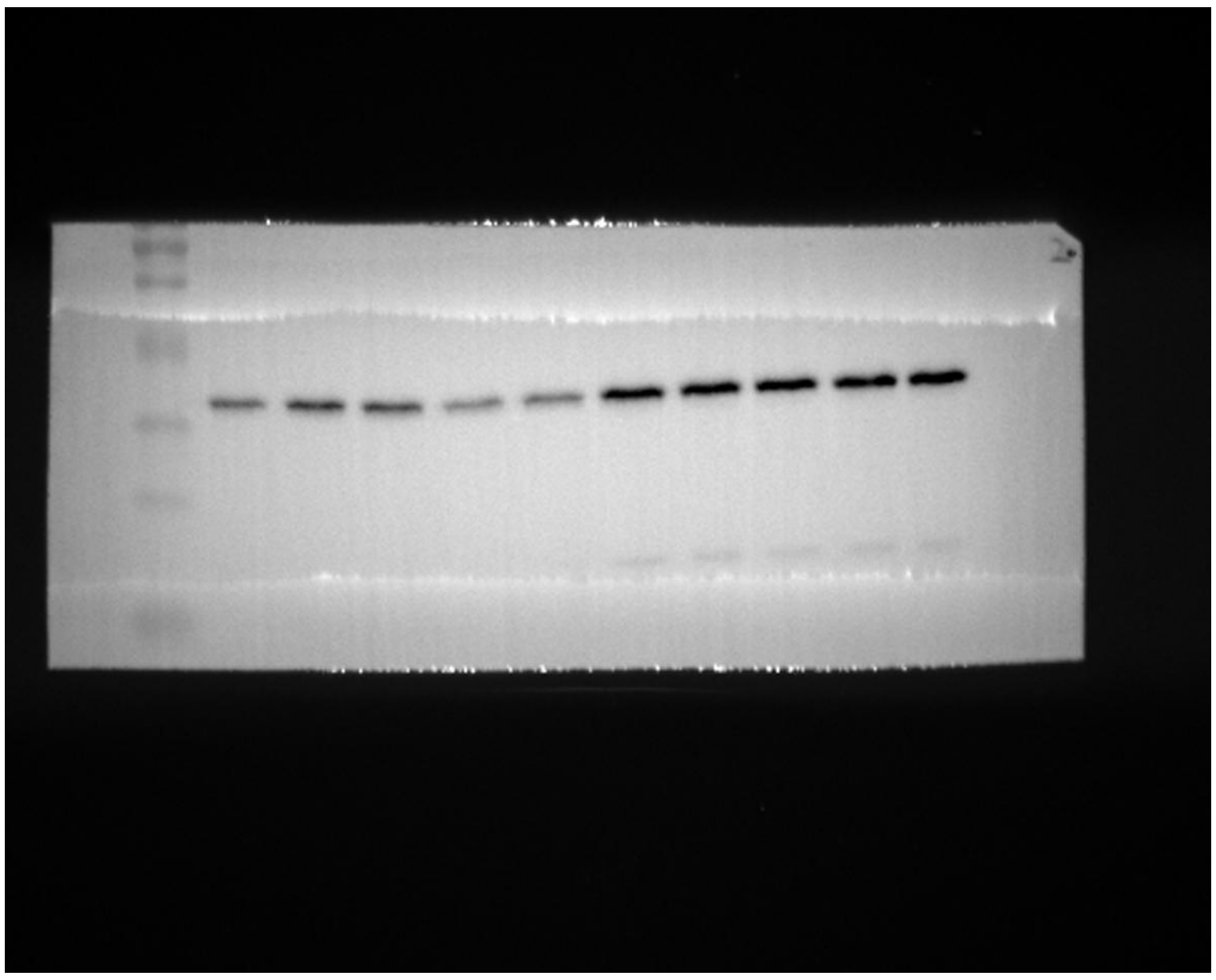

Figure3A HIF1α

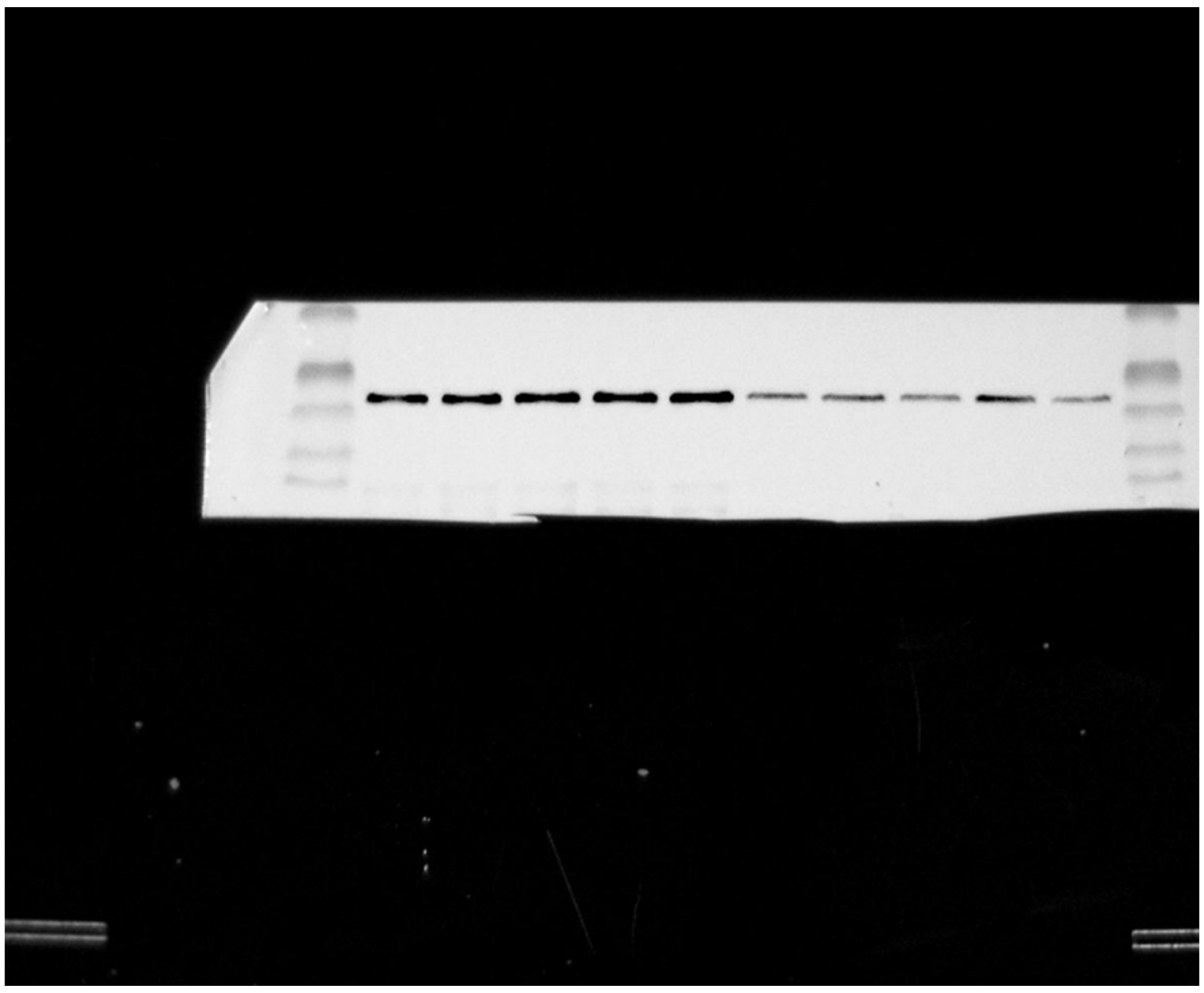

Figure 3A TLR 4

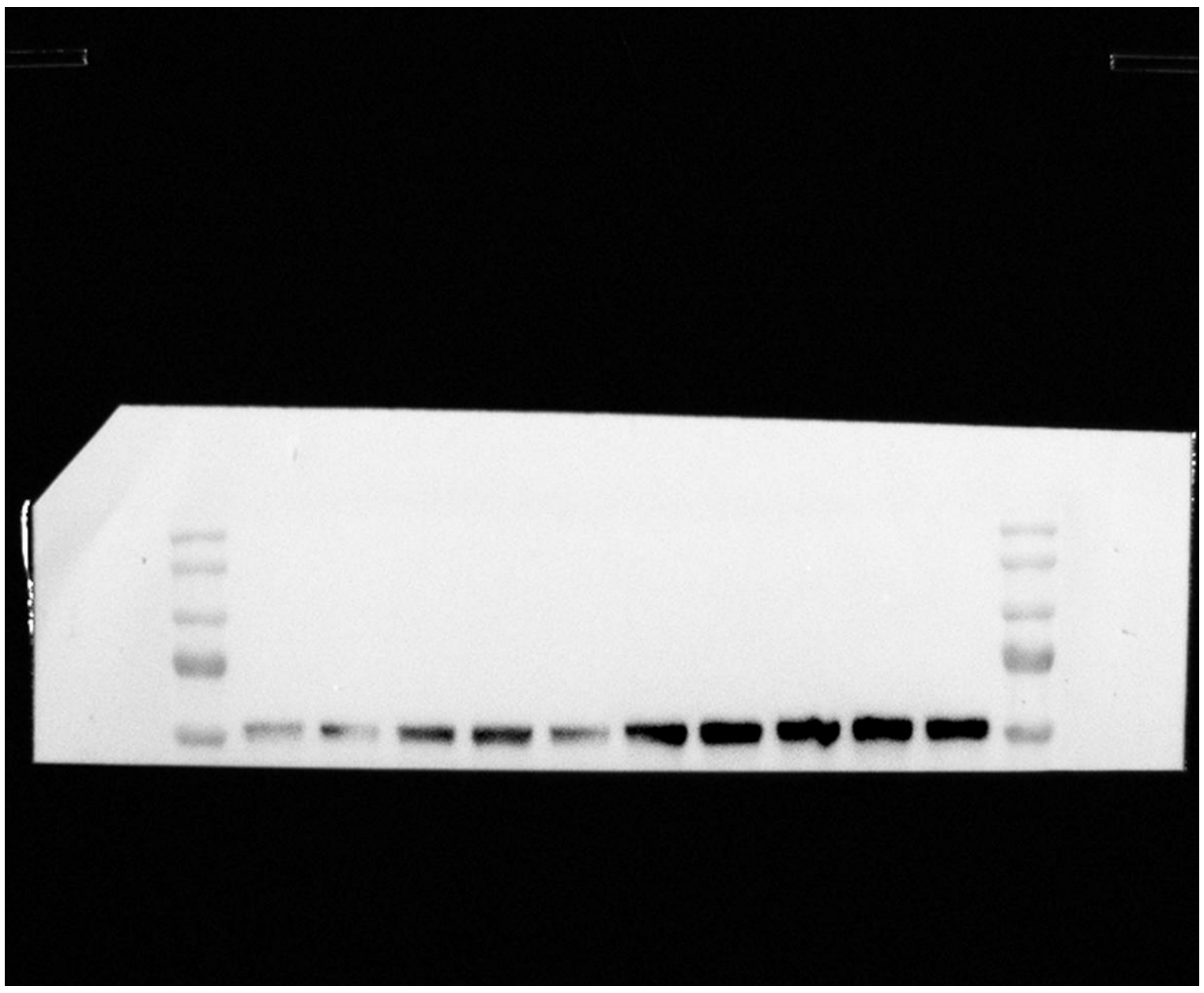

Figure 3A G A P D H

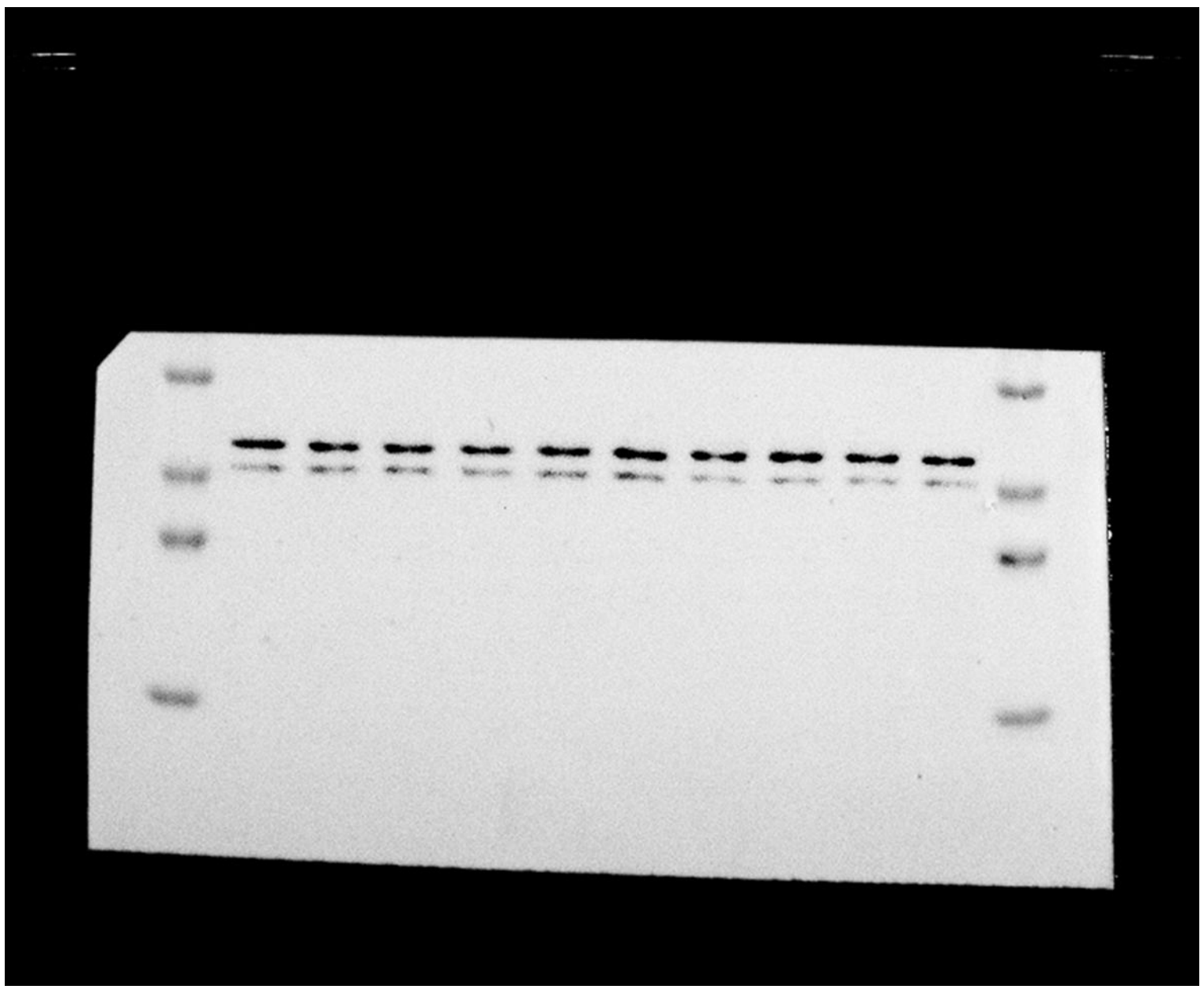

Figure3A NF-κB

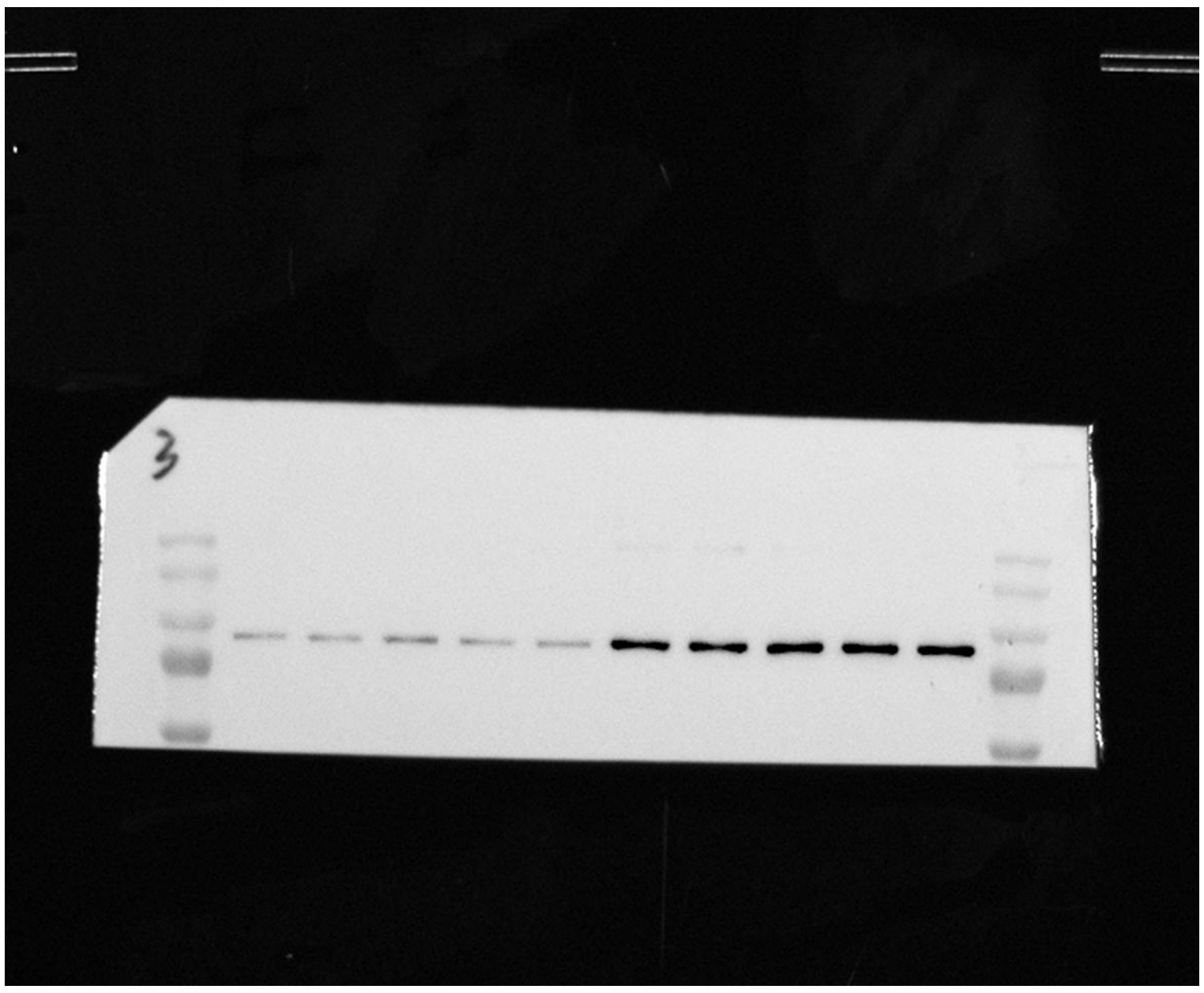

Supplement: Supplementary file 1 [file pone.0355467.s001.pdf]
